# Progression of kidney injury with the combination of vancomycin and piperacillin-tazobactam or cefepime in sepsis-associated acute kidney injury

**DOI:** 10.3389/fneph.2022.995358

**Published:** 2022-10-20

**Authors:** Kaylee Whitenack, Michael L. Behal, Melissa L. Thompson Bastin, Juan C. Aycinena, Paul M. Adams, Alexander H. Flannery

**Affiliations:** ^1^ Department of Pharmacy Practice and Science, University of Kentucky College of Pharmacy, Lexington, KY, United States; ^2^ Department of Pharmacy Services, University of Kentucky HealthCare, Lexington, KY, United States; ^3^ Division of Nephrology, Bone & Mineral Metabolism, Department of Internal Medicine, University of Kentucky College of Medicine, Lexington, KY, United States

**Keywords:** vancomycin, piperacillin, tazobactam, nephrotoxicity, acute kidney injury, sepsis

## Abstract

**Introduction:**

The combination of vancomycin/piperacillin-tazobactam is associated with increases in serum creatinine compared to other antibiotic combinations in the treatment of infections for hospitalized patients. However, the available literature is limited to the study of incident acute kidney injury (AKI). The combination has not been evaluated in patients with AKI already present and the degree to which the trajectory of AKI is influenced by this combination is unknown.

**Methods:**

This was a single center, retrospective cohort study of adult patients with sepsis and AKI present on admission prescribed a combination of vancomycin with either piperacillin-tazobactam or cefepime within the first 3 days of admission. The primary outcome was maximum serum creatinine observed within days 2-7 of the hospital stay. Subsequent kidney outcomes were evaluated at one week and hospital discharge.

**Results:**

Of 480 patients with sepsis and AKI who met inclusion criteria, 288 (60%) received vancomycin/piperacillin-tazobactam, and 192 (40%) received vancomycin/cefepime. Patients were well-matched on clinical factors, including severity of illness, stage of AKI, exposure to other nephrotoxins, and durations of antimicrobial therapy. There were no differences in AKI trajectory during the first week as assessed by maximum serum creatinine (2.1 (1.4-3.5) mg/dl vs. 2.1 (1.4-3.0) mg/dl; p=0.459) and AKI progression (24.0% vs. 23.4%; p=0.895). No differences were observed with other kidney related outcomes, including the need for dialysis (14.6% vs. 13.0%; p=0.628) or major adverse kidney events at hospital discharge (48.3% vs. 47.9%; p=0.941).

**Conclusions:**

In patients with sepsis and AKI, the combination of vancomycin/piperacillin-tazobactam compared to vancomycin/cefepime was not associated with higher serum creatinine values or AKI progression in the week following ICU admission.

## Introduction

In recent years, several observational studies have suggested the combination of vancomycin and piperacillin-tazobactam is associated with increased acute kidney injury (AKI) compared to the combination of vancomycin with other beta-lactam antibiotics such as cefepime or meropenem (1–3). The entirety of data available involve incident AKI; thus patients with AKI are often excluded. However, vancomycin combined with a beta-lactam is a common empiric antibiotic prescription for patients with sepsis, and up to 42% of patients with sepsis will present with concurrent AKI, often termed sepsis-associated AKI (SA-AKI) (4). Thus, consideration to potential nephrotoxicity from these antibiotic combinations is a common clinical challenge when caring for patients with SA-AKI.

These data appear to be impacting clinical practice, with a recent survey indicating a majority of clinicians are less likely to recommend combination therapy with vancomycin and piperacillin-tazobactam in a patient with preexisting AKI (5). The downstream effects of alternative combination regimens in SA-AKI include concerns with neurotoxicity (cefepime), lack of anaerobic coverage (cefepime), and antibiotic stewardship (meropenem) (6). Further, kidney function at discharge for patients with SA-AKI is predictive of long-term kidney complications (7), and it is unknown how the potential nephrotoxicity of these different antibiotic combinations may impact the progression, or resolution, of SA-AKI out to hospital discharge. Accordingly, we sought to evaluate the progression of kidney injury in SA-AKI patients prescribed the combination of vancomycin and piperacillin-tazobactam compared to vancomycin and cefepime.

## Materials and methods

### Study design

This was a single center, retrospective cohort study of critically ill adult patients admitted to the medical intensive care unit (ICU) of a tertiary care hospital with SA-AKI over a 7.5-year period (January 1, 2013-July 31, 2020). Patients were initially considered if they were ≥ 18 years of age and presented with both sepsis (including septic shock) and AKI on admission. Patients with sepsis or septic shock were identified based on International Classification of Diseases (ICD)-9 and ICD-10 codes, while AKI was classified as stage 1 AKI or higher using the serum creatinine component of the Kidney Disease: Improving Global Outcomes (KDIGO) criteria (8). Exclusion criteria included: transfer from an outside hospital, death within 24 hours of admission, or end-stage kidney disease. If multiple ICU admissions were identified during the time period, only the first ICU encounter was included in the analysis.

Patient demographics, comorbidities, and severity of illness measures were extracted from the Enterprise Data Warehouse at the University of Kentucky. Comorbidities were identified based on ICD-9 and -10 codes and severity of illness using the Sequential Organ Failure Assessment (SOFA) score, which was calculated electronically upon data extraction (9). Baseline kidney function was assessed using the most recent serum creatinine in the six months prior to admission, when available. The Chronic Kidney Disease Epidemiology Collaboration (CKD-EPI) equation was used to calculate estimated glomerular filtration rate (eGFR) (10).

Patients were classified into two groups based on their receipt of vancomycin in combination with either piperacillin-tazobactam or cefepime within the first 72 hours of ICU admission. Given that vancomycin is often dosed intermittently in the setting of AKI, vancomycin exposure was defined as one or more doses of vancomycin received on day 1 or 2 of ICU admission. Further, patients must have received at least 2 consecutive days (within the first 72 hours) of piperacillin-tazobactam or cefepime to be classified into those respective groups. In order to strictly define the exposure of interest, patients were excluded if they received more than one beta-lactam antibiotic within the first 72 hours of ICU admission. Antibiotic prescribing was at the discretion of the treating medical team; no formal protocols existed to direct selection of either piperacillin-tazobactam or cefepime over the other during the time period evaluated. Due to the minimal number of patients that received solely the combination of vancomycin and meropenem, we excluded patients who were given this combination in order to focus the comparison on piperacillin/tazobactam and cefepime combination therapy with vancomycin.

The primary outcome compared between groups was the maximum serum creatinine value between days 2 and 7. This outcome was selected to evaluate the patient’s kidney function within the first week of ICU admission for as long as possible, given the competing event of mortality common in sepsis research. Secondary outcomes included AKI progression within the first 7 days (defined as an advancement in KDIGO stage from admission), composite of Acute Kidney Disease (AKD) based on the Acute Disease Quality Initiative consensus definition (11) and death within 7 days, requirement for kidney replacement therapy (KRT) within 7 days, ICU mortality, ICU and hospital length of stay, and Major Adverse Kidney Events (MAKE) assessed at hospital discharge, a composite of death, need for KRT, or decrease in eGFR ≥25% from baseline (12). The study was approved by the Institutional Review Board (IRB) at the University of Kentucky with patient follow-up for all outcomes truncated at discharge. The methods and findings are reported per the Strengthening the Reporting of Observational Studies in Epidemiology (STROBE) guidelines for cohort studies (13).

### Statistical analysis

Our prior work in this population suggested a maximum serum creatinine of 3 ± 2 mg/dl in the first week for critically ill SA-AKI patients. Assuming that the combination of vancomycin and piperacillin-tazobactam would result in a 20% higher maximum serum creatinine in the first week compared to the combination of vancomycin and cefepime, 176 patents in each group (n=352 total) would be necessary for 80% power with α=0.05.

Categorical data are presented as counts along with percentages and compared using a chi-square test. Continuous data are presented using medians (interquartile range) and compared between groups using the Wilcoxon rank-sum test. In those cases where no baseline serum creatinine was documented prior to admission, multiple imputations were conducted using SAS 9.4 Proc MI (SAS Institute Inc) including the variables age, sex, race, diabetes, and hypertension, full conditional specification, with 50 imputed data sets and the average creatinine value from the 50 imputations was used to assess AKI staging for inclusion in the cohort and further classification (14, 15). A sensitivity analysis of maximum serum creatinine and AKI progression was conducted in the subset of patients with a documented (non-imputed) baseline serum creatinine to evaluate the potential influence of imputation on the findings.

## Results

**Figure 1:**
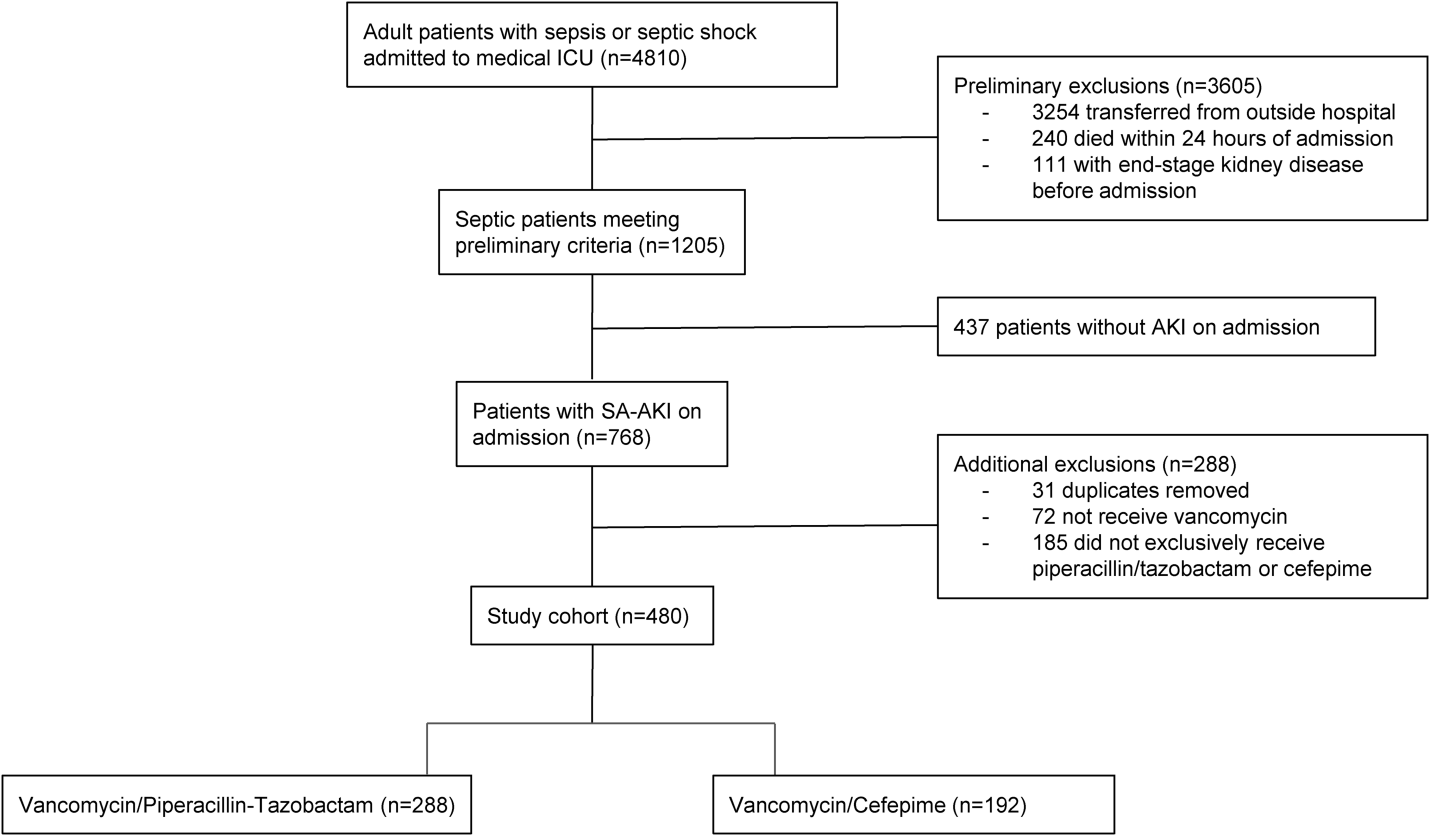
Flow Diagram for Study Inclusion and Exclusion.

From January 2013 to July 2020, 4,810 patients were admitted to the medical ICU with sepsis. Following the subsequent inclusion and exclusion criteria applied (**Figure 1**), 480 patients with SA-AKI were included for final evaluation with exclusive receipt of either combination vancomycin/piperacillin-tazobactam (n=288) or vancomycin/cefepime (n=192) within the first 72 hours of ICU admission.

Patient demographics are shown in **Table 1**. The two cohorts were well-matched at baseline without any significant differences in terms of demographics, comorbidities, or severity of illness. The cohort was predominantly Caucasian, with slightly more males than females, and with a typical comorbidity pattern observed in medically critically ill patients. A documented baseline creatinine was available for 230/480 (47.9%) of patients in the cohort with a similar degree of missingness between groups (p=0.709). Acuity of illness was high as demonstrated by SOFA scores and with over three-quarters of the cohort exhibiting a requirement for vasopressors within 48 hours of ICU admission. Importantly, serum creatinine, blood urea nitrogen, and KDIGO AKI stage were similar between groups during the first 2 days of admission. A similar number of patients received concurrent aminoglycosides in each group. The duration of antimicrobial therapy, assessed within the first 7 days, was similar between the two groups.

**Table 1:** Patient Demographics.

Demographic	Vancomycin & Piperacillin-Tazobactam(n=288)	Vancomycin & Cefepime(n=192)	p-value
Age (years)	57 (45-66)	57 (45-66)	0.849
Sex (% males)	158 (54.9%)	108 (56.3%)	0.764
Race (% white)	263 (91.3%)	168 (87.5%)	0.270
Height (cm)	173 (163-178)	173 (164-180)	0.639
Weight (kg)	80 (68-93)	82 (68-104)	0.230
Diabetes (%)	100 (34.7%)	66 (34.4%)	0.938
Liver disease (%)	92 (31.9%)	61 (31.8%)	0.968
Chronic kidney disease (%)	70 (24.3%)	42 (21.9%)	0.537
Hypertension (%)	176 (61.1%)	122 (63.5%)	0.591
Heart failure (%)	64 (22.2%)	47 (24.5%)	0.566
Coronary artery disease (%)	98 (34.0%)	58 (30.2%)	0.381
SOFA	10 (6-13)	9 (6-13)	0.422
Admission lactate (mmol/l)	1 (0.4-3.8)	1.5 (0.5-3.7)	0.106
Vasopressor requirement within 48 hr of admission (%)	226 (78.5%)	146 (76.0%)	0.532
Baseline eGFR (ml/min/1.73m^2^)	86 (66-105)	83 (67-105)	0.958
KDIGO stage of AKI:			0.667
Stage 1	107 (37.2%)	75 (39.1%)	–
Stage 2	72 (25.0%)	52 (27.1%)	–
Stage 3	109 (37.9%)	65 (33.9%)	–
Maximum creatinine (mg/dl)			
Day 1	2.3 (1.5-3.4)	2.2 (1.5-3.3)	0.494
Day 2	2.0 (1.3-3.2)	2.0 (1.4-2.9)	0.310
Maximum BUN (mg/dl)			
Day 1	38 (26-65)	37 (25-59)	0.805
Day 2	39 (24-60)	36 (25-59)	0.700
Received aminoglycosides within 72 hours of admission (%)	50 (17.4%)	31 (16.2%)	0.728
Number of days receiving vancomycin within first week (d)	3 (1-4)	3 (2-5)	0.177
Number of days receiving beta-lactam^a^ within first week (d)	4 (3-6)	5 (3-7)	0.080

a beta-lactam refers to piperacillin-tazobactam or cefepime, respectively.

SOFA, Sequential Organ Failure Assessment score; eGFR, estimated glomerular filtration rate; KDIGO, Kidney Disease: Improving Global Outcomes; BUN, blood urea nitrogen.

**Figure 2:**
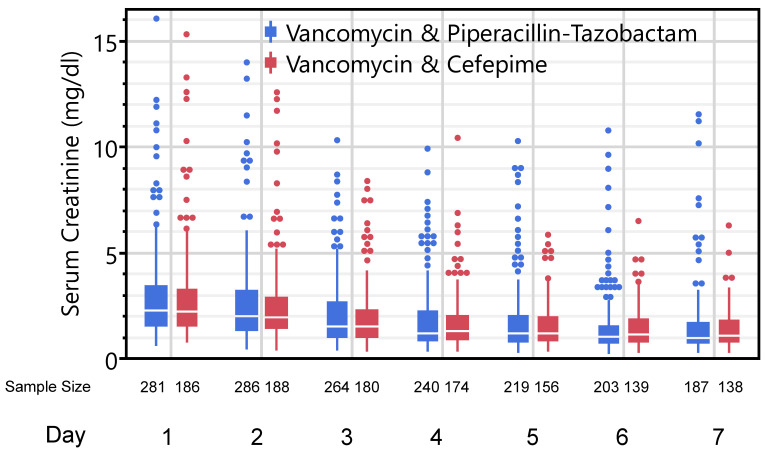
Serum Creatinine in the 7 Days Following Therapy Initiation.

The primary outcome of maximum serum creatinine observed on days 2-7 was similar between groups: 2.1 (1.4-3.5) mg/dl vs. 2.1 (1.4-3.0) mg/dl in the vancomycin/piperacillin-tazobactam and vancomycin-cefepime groups, respectively (p=0.459). Daily serum creatinine values between the two groups over the course of the first week are displayed in **Figure 2**. There was also no difference in AKI progression to the next KDIGO stage in the first week between the two groups: 24.0% vs. 23.4% (p=0.895). Patients in both groups exhibited similar lengths of stay and ICU mortality. Finally, there were no differences in kidney-specific secondary outcomes between the two groups including the composite of death or AKD, requirement for KRT within 7 days, or MAKE assessed at discharge. Complete secondary outcomes are reported in **Table 2**. In sensitivity analyses only considering those cases with a documented (non-imputed) baseline serum creatinine, there were no differences in maximum creatinine (2.1 (1.3-3.4) vs. 2.0 (1.5-2.8); p=0.775) or AKI progression (23.5% vs. 28.7%; p=0.375) in the vancomycin/piperacillin-tazobactam and vancomycin-cefepime groups, respectively.

**Table 2:** Study Outcomes.

Outcome	Vancomycin & Piperacillin-Tazobactam(n=288)	Vancomycin & Cefepime(n=192)	p-value
Maximum serum creatinine Days 2-7 (mg/dl)	2.1 (1.4-3.5)	2.1 (1.4-3.0)	0.459
AKI progression (%)	69 (24.0%)	45 (23.4%)	0.895
Composite of AKD and death within 7 days (%)	129 (44.8%)	92 (47.9%)	0.501
Requirement for KRT within 7 days (%)	42 (14.6%)	25 (13.0%)	0.628
ICU mortality (%)	69 (24.0%)	46 (24.0%)	1.000
ICU length of stay (d)	3.0 (0.3-7.1)	2.9 (0.3-7.6)	0.884
Hospital length of stay (d)	9.2 (4.9-16.5)	11.9 (5.7-20.6)	0.062
MAKE at hospital discharge (%)	139 (48.3%)	92 (47.9%)	0.941
Death (%)	93 (32.3%)	60 (31.3%)	0.810
KRT at discharge (%)	6 (2.1%)	5 (2.6%)	0.709
Reduced eGFR at discharge (%)	40 (13.9%)	27 (14.1%)	0.957

AKI, acute kidney injury; AKD, acute kidney disease; KRT, kidney replacement therapy; ICU, intensive care unit; MAKE, major adverse kidney event; eGFR, estimated glomerular filtration rate.

## Discussion

Our study observed no difference in kidney-specific or other secondary outcomes, including the maximum serum creatinine in the first week and AKI progression, between vancomycin/piperacillin-tazobactam and vancomycin/cefepime combination therapy in patients with SA-AKI. This cohort is unique as the first to evaluate these combinations’ potential for nephrotoxicity in the setting of existing AKI. Prior literature studying the potential nephrotoxicity of vancomycin/piperacillin-tazobactam has excluded AKI at baseline (i.e. the start date of vancomycin/piperacillin-tazobactam prescription) in order to evaluate incident or new AKI while on therapy (1, 16). While this is necessary to study a particular question about the potential nephrotoxicity of vancomycin/piperacillin-tazobactam (i.e. incident AKI), we also suggest that it excludes a large proportion of patients prescribed this combination in clinical practice. We studied a population in sepsis that is universally exposed to antibiotic therapy by nature of the condition but also commonly presents with AKI in over 40% of cases (4).

While vancomycin is a well-documented nephrotoxin warranting therapeutic drug monitoring (17, 18), recent data have consistently shown an association between the combination of vancomycin/piperacillin-tazobactam (compared to vancomycin combined with other beta-lactam antibiotics) and AKI assessed using serum creatinine (1, 16). Precise mechanisms for this observation continue to be debated, particularly given that piperacillin-tazobactam is not commonly considered a nephrotoxin as monotherapy. Most of the AKI assessments suggesting nephrotoxicity from the combination of vancomycin/piperacillin-tazobactam conducted in retrospective observational studies are based on serum creatinine. In a secondary analysis of the Sapphire study, kidney stress markers, specifically the product of tissue inhibitor of metalloproteinase-2 (TIMP-2) and insulin-like growth factor binding-protein 7 (IGFBP7), were higher in patients receiving the combination of vancomycin/piperacillin-tazobactam compared to either agent alone (19, 20). However, debate remains over the specific mechanisms of nephrotoxicity from the combination, and whether the phenomenon observed is due to impaired tubular secretion of creatinine, or pseudo-nephrotoxicity, without any injury to the kidney (21).

Both piperacillin and tazobactam are substrates of organic anion transporter (OAT) 1 and 3 and may compete with serum creatinine secretion from the basolateral side of the nephron (22, 23). There are also data that vancomycin may reduce the expression of these OATs, subsequently decreasing creatinine secretion and increasing serum creatinine concentrations (24). OAT1 and 3 are downregulated in AKI, presumed to be the case in our cohort of patients with SA-AKI, which may alter the interplay of piperacillin-tazobactam, vancomycin, and creatinine secretion if the pseudo-nephrotoxicity hypothesis is true (25). While this is hypothesis-generating only as we did not measure OATs, translational rat models have shown no decrease in GFR or urinary biomarkers of kidney injury when vancomycin and piperacillin-tazobactam are used together (26). Regardless of the potential mechanism for a creatinine elevation without kidney injury, we did not observe any impact on kidney function in the subsequent week following antibiotic initiation, nor at hospital discharge, as assessed with multiple endpoints.

The combination of vancomycin and piperacillin-tazobactam is one of the most commonly used empiric regimens for sepsis in hospitalized patients (27). Importantly, the number of studies published on the potential nephrotoxicity of vancomycin and piperacillin-tazobactam have influenced prescribing patterns, which may have downstream implications. In a survey of infectious disease and critical care pharmacists, 89% of respondents reported a belief that the combination of vancomycin and piperacillin-tazobactam resulted in an increased probability of developing AKI compared with vancomycin alone or in combination with other beta-lactam antibiotics (5). Of those respondents, 64% reported they less frequently recommend the combination of vancomycin and piperacillin-tazobactam in a patient with existing AKI (5). This would appear to complicate the antibiotic selection for patients with sepsis and AKI. Clinically, alternative regimens would include vancomycin in combination with either cefepime or meropenem, which each have issues of their own. Cefepime is associated with encephalopathy and neurotoxicity in 3-23% of patients, particularly in the setting of reduced kidney function; nearly 90% of patients with neurotoxicity attributed to cefepime have reduced kidney function (6, 28–33). Cefepime also does not provide anaerobic coverage as may be desired in empiric antibiotic therapy, requiring additional antibiotic exposure which may increase cost, volume intake, and nursing time. Meropenem as empiric therapy for sepsis carries significant antimicrobial stewardship concerns due to the potential development of resistance to precious antibiotic resources (34). Although each combination undoubtedly comes with some set of risks and benefit, our study adds additional data for the clinician to consider, and shows similar kidney outcomes in patients with SA-AKI and suggests the combination of vancomycin/piperacillin-tazobactam may not need to be avoided in this population in attempts to spare kidney function.

This study contains several unique strengths. It is the first to our knowledge to address the issue of vancomycin/piperacillin-tazobactam nephrotoxicity in the setting of existing kidney injury, a scenario where combination antibiotic therapy is routinely utilized when treating patients with sepsis and AKI. As noted, prior evaluations of the potential nephrotoxicity of vancomycin and piperacillin-tazobactam have excluded AKI patients, including studies from different time periods conducted at our center (35, 36), which makes the AKI patient population extremely under-represented in this line of inquiry regarding potential nephrotoxicity of antibiotic combinations. Second, we used strict beta-lactam inclusion definitions, which resulted in included patients having unique and well-defined antibiotic combinations, without overlapping beta-lactam antibiotics to confound the study. Third, we evaluated several different kidney endpoints at various time intervals in order to evaluate safety of this combination in patients presenting with AKI.

This study is also subject to several limitations. First, the single center, retrospective nature limits the sample size and potential generalizability to other centers. Second, we did not measure GFR nor any notable biomarkers of kidney injury or function to phenotype AKI; our results were solely based on serum creatinine, which has well-identified limitations (37). We also did not capture urine output which may have differentiated the KDIGO grade transition independently of serum creatinine. Third, we limited our study to critically ill patients, and the combination of vancomycin and piperacillin-tazobactam may have differential effects on kidney function in critically ill vs. non-critically ill patients (1). Fourth, we were unable to quantify exposure, including dosing and therapeutic drug monitoring for all drugs in question, which may play a role in assessing any toxicity. Fifth, we did not conduct a time-to-event analysis to a meaningful event such as AKI progression or recovery, although we also acknowledge that death likely represents a significant competing event in this SA-AKI population for this type of approach. Such an approach, if carefully considered, may be beneficial in future work. Finally, although the groups appeared to be well-balanced, we cannot eliminate residual confounding in a study of this nature.

## Conclusion

In patients with SA-AKI, the combination of vancomycin/piperacillin-tazobactam compared to vancomycin/cefepime was not associated with higher serum creatinine values or AKI progression in the week following ICU admission. Other kidney-related outcomes measured out to hospital discharge were similar between groups. This suggests use of vancomycin/piperacillin-tazobactam during existing SA-AKI may need not necessarily be avoided for nephrotoxicity concerns when considering the safety profiles and limitations of alternatives.

## Data availability statement

The raw data supporting the conclusions of this article will be made available by the authors, without undue reservation.

## Ethics statement

The studies involving human participants were reviewed and approved by University of Kentucky Institutional Review Board. Written informed consent for participation was not required for this study in accordance with the national legislation and the institutional requirements.

## Author contributions

KW and AF are responsible for developing the study question and design, MB contributed to data acquisition and cleaning, AF is responsible for statistical analysis, KW and AF wrote the initial draft of the manuscript, and MB, MTB, JA, and PA provided critical insights and analysis. All authors have read and approve of the final version of the manuscript.

## Funding

AF is supported by grant K23DK128562. The project described was supported by the NIH National Center for Advancing Translational Sciences through grant number UL1TR001998. The content is solely the responsibility of the authors and does not necessarily represent the official views of the NIH.

## Conflict of interest

The authors declare that the research was conducted in the absence of any commercial or financial relationships that could be construed as a potential conflict of interest.

## Publisher’s note

All claims expressed in this article are solely those of the authors and do not necessarily represent those of their affiliated organizations, or those of the publisher, the editors and the reviewers. Any product that may be evaluated in this article, or claim that may be made by its manufacturer, is not guaranteed or endorsed by the publisher.
